# Supplementary figures and images for: Machine learning identifies signatures of host adaptation in the bacterial pathogen Salmonella enterica
Source: PLoS Genet. 2018 May 8;14(5):e1007333. doi: 10.1371/journal.pgen.1007333 (PMC5940178; doi:10.1371/journal.pgen.1007333)

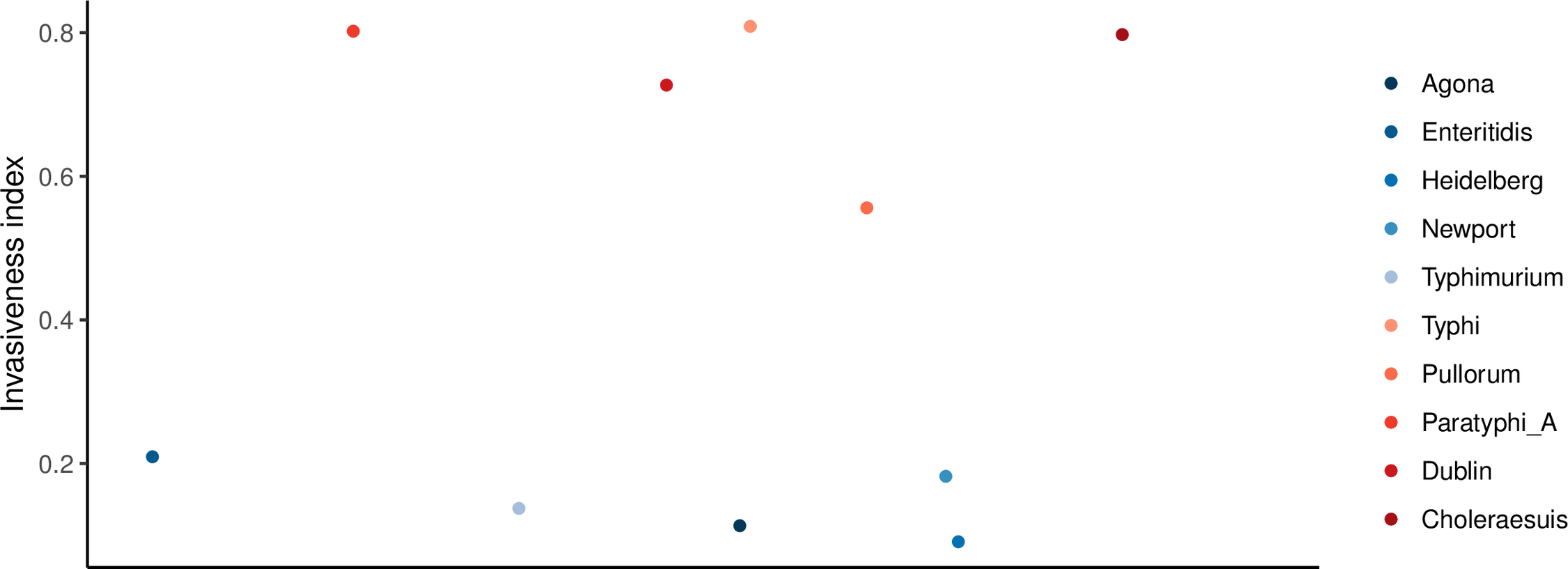

Supplement: S1 Fig — (TIF) [file pgen.1007333.s001.tif]

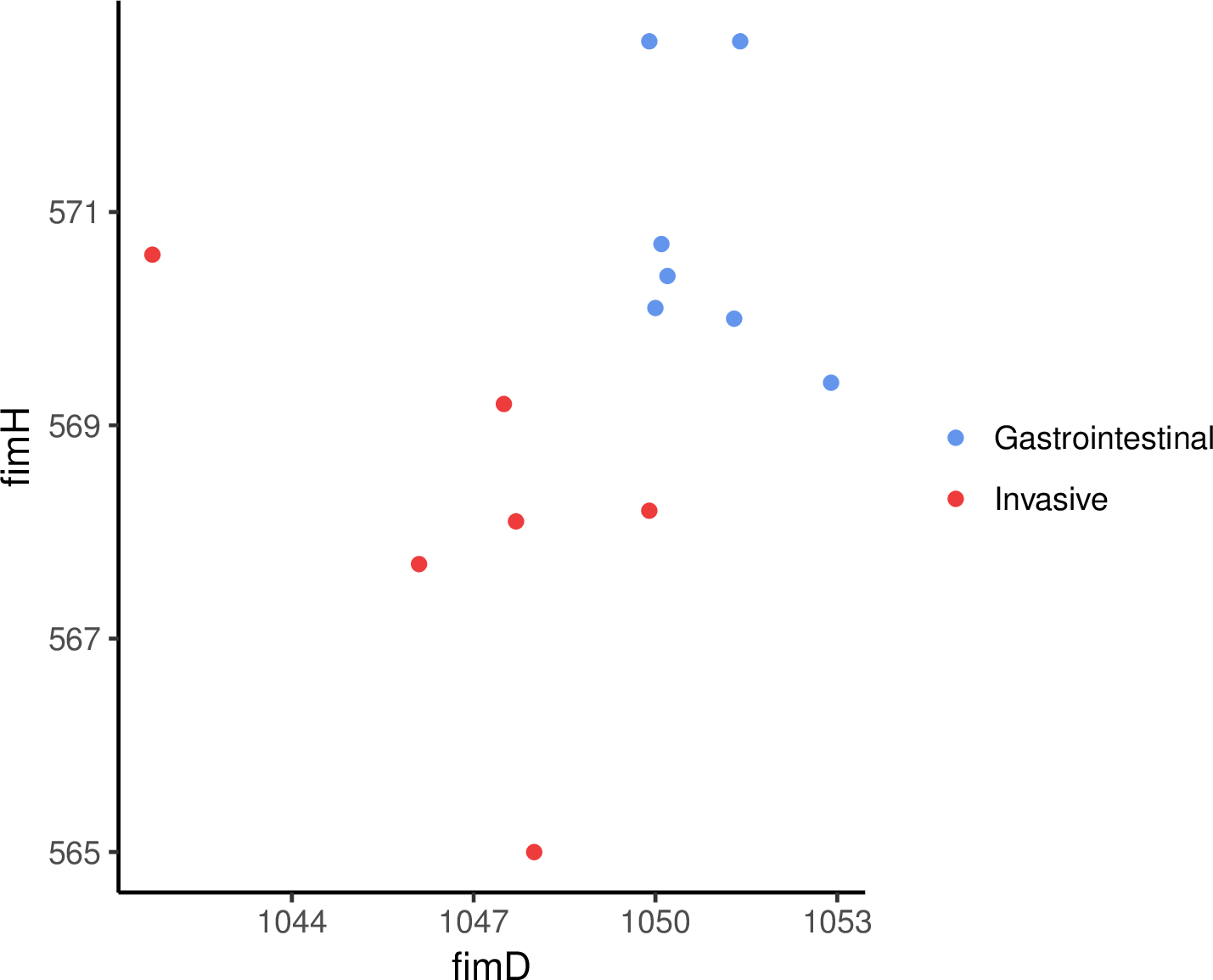

Supplement: S2 Fig — (TIF) [file pgen.1007333.s002.tif]

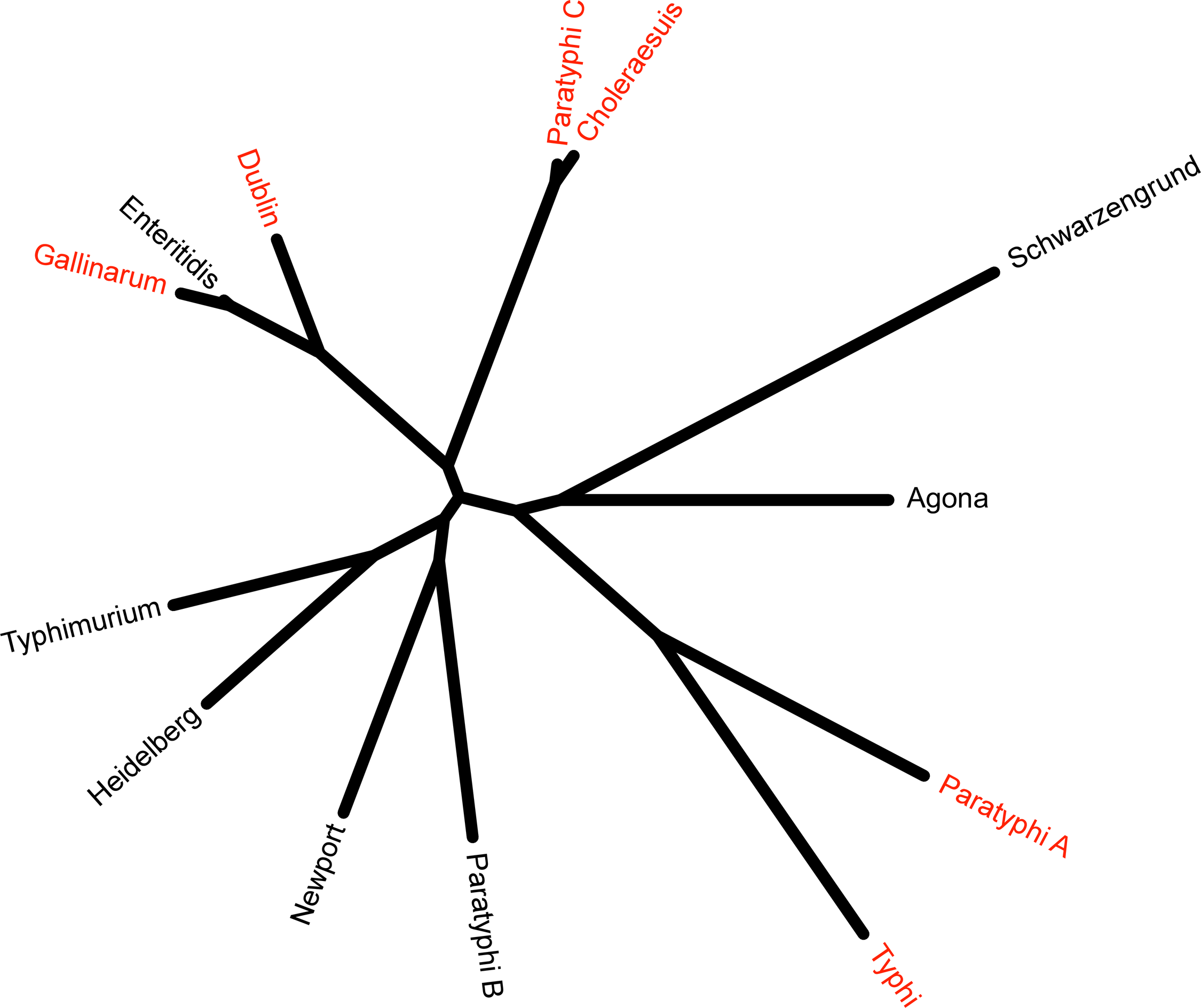

Supplement: S3 Fig — The tree was constructed in RAxML using a core gene alignment produced by Roary. Invasive serovars are highlighted in red. (TIF) [file pgen.1007333.s003.tif]

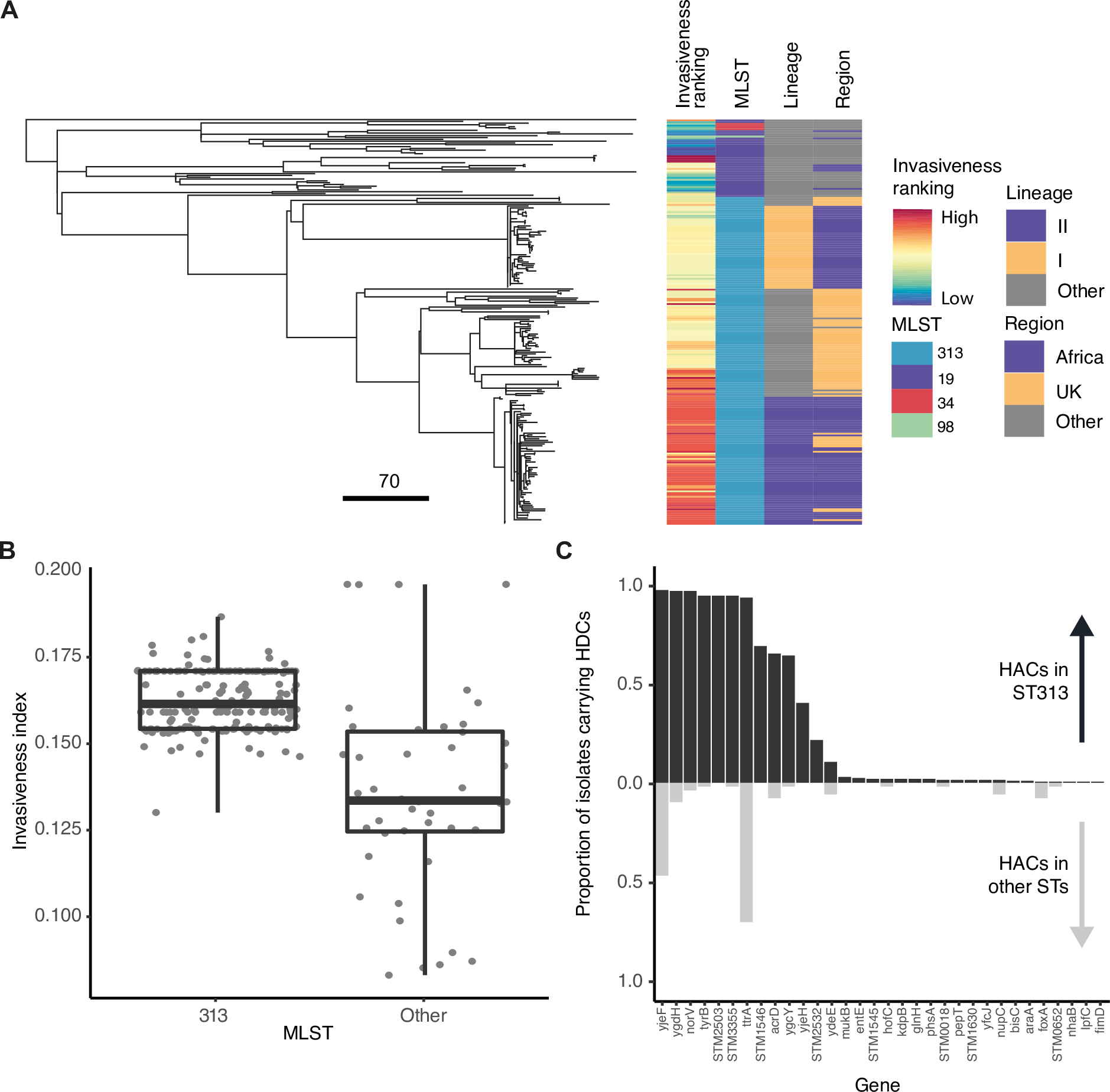

Supplement: S4 Fig — A: RAxML tree of all ST313 isolates included in the study, annotated with invasiveness ranking and lineage. B: Invasiveness index for all ST313 isolates. C: Proportion of isolates carrying HACs in ST313 compared to other sequence types. (TIF) [file pgen.1007333.s004.tif]

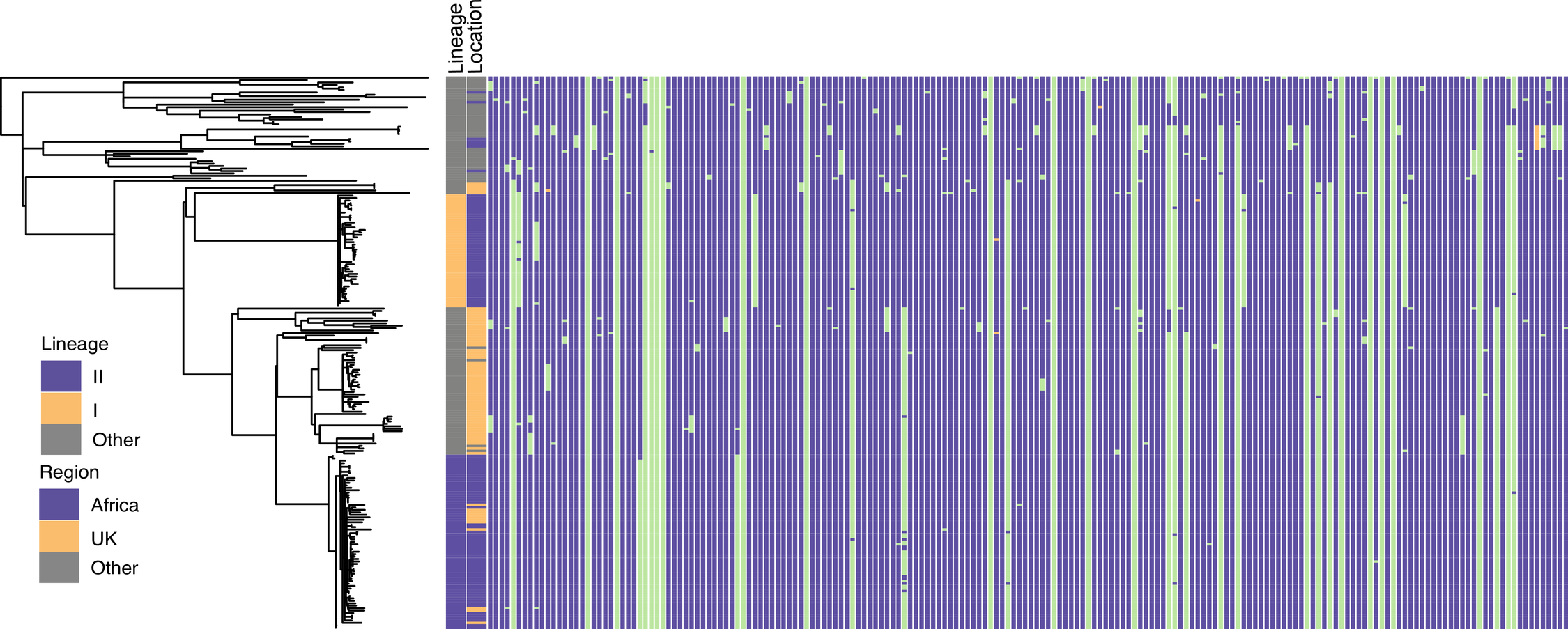

Supplement: S5 Fig — Genes for which homology to the reference sequence was not detected (usually due to extreme truncation) are marked in orange. (TIF) [file pgen.1007333.s005.tif]
